# Paired involvement of human-specific Olduvai domains and *NOTCH2NL* genes in human brain evolution

**DOI:** 10.1007/s00439-019-02018-4

**Published:** 2019-05-13

**Authors:** Ian T. Fiddes, Alex A. Pollen, Jonathan M. Davis, James M. Sikela

**Affiliations:** 1 10X Genomics, Pleasanton, CA USA; 2 Department of Neurology and the Eli and Edythe Broad Center for Regeneration Medicine and Stem Cell Research at the University of California, San Francisco, San Francisco, CA USA; 3 Department of Biochemistry and Molecular Genetics, Human Medical Genetics and Genomics Program and Neuroscience Program, University of Colorado School of Medicine, Aurora, CO 80045 USA

## Abstract

Sequences encoding Olduvai (DUF1220) protein domains show the largest human-specific increase in copy number of any coding region in the genome and have been linked to human brain evolution. Most human-specific copies of Olduvai (119/165) are encoded by three *NBPF* genes that are adjacent to three human-specific *NOTCH2NL* genes that have been shown to promote cortical neurogenesis. Here, employing genomic, phylogenetic, and transcriptomic evidence, we show that these *NOTCH2NL*/*NBPF* gene pairs evolved jointly, as two-gene units, very recently in human evolution, and are likely co-regulated. Remarkably, while three *NOTCH2NL* paralogs were added, adjacent Olduvai sequences hyper-amplified, adding 119 human-specific copies. The data suggest that human-specific Olduvai domains and adjacent *NOTCH2NL* genes may function in a coordinated, complementary fashion to promote neurogenesis and human brain expansion in a dosage-related manner.

**Electronic supplementary material:**

The online version of this article (10.1007/s00439-019-02018-4) contains supplementary material, which is available to authorized users.

## Introduction

The increasing availability of primate genomic data is providing a unique opportunity to identify lineage-specific sequence differences among human and other primates. As part of these efforts, genomic factors are beginning to be uncovered that potentially contributed to the evolutionary expansion of the human brain (Sikela 2006; O’Bleness et al. 2012a). While there are a number of genomic mechanisms that may have been critical to brain expansion, several candidate sequences have been reported that involve human-specific gene duplications (Dennis et al. 2012; Florio et al. 2015). A number of these sequences map to 1q21, a region on chromosome 1 that is highly enriched for human lineage-specific gene duplications (Fortna et al. 2004; Dumas et al. 2007). This finding should not be unexpected, as unique evolutionary features of the region have been known to cytogeneticists for decades: it is the site of a human-specific pericentric inversion and is adjacent to a human-specific C-band (Yunis and Prakash 1982).

Among the duplicated genes and coding regions in 1q21 that have been associated with brain evolution, the most dramatically changed are those specifying Olduvai protein domains (formerly DUF1220) (Popesco et al. 2006; Sikela and van Roy 2017). Encoded primarily by the *NBPF* gene family (Vandepoele et al. 2005), Olduvai sequences have undergone the largest human lineage-specific increase in copy number of any coding region in the genome (~ 300 total copies of which ~ 165 are human-specific) (Popesco et al. 2006; O’Bleness et al. 2012a, b). They have been implicated, in a dose-dependent manner, in brain size [both evolutionarily as well as within the human population (Dumas et al. 2012; Keeney et al. 2014; Zimmer and Montgomery 2015)] and cognitive function (Davis et al. 2014a), and have been shown to promote proliferation in neural stem cells (Keeney et al. 2015). In addition, variation in Olduvai copy number has been associated with cognitive disease: autism, schizophrenia, microcephaly and macrocephaly (Dumas et al. 2012; Davis et al. 2014b, 2015, 2019; Quick et al. 2015). This ability to potentially confer both beneficial and detrimental effects has led to the proposal that the Olduvai family may constitute a cognitive genomic trade-off specific to the human lineage (Dumas and Sikela 2009; Sikela and Quick 2018).

**Fig. 1:**
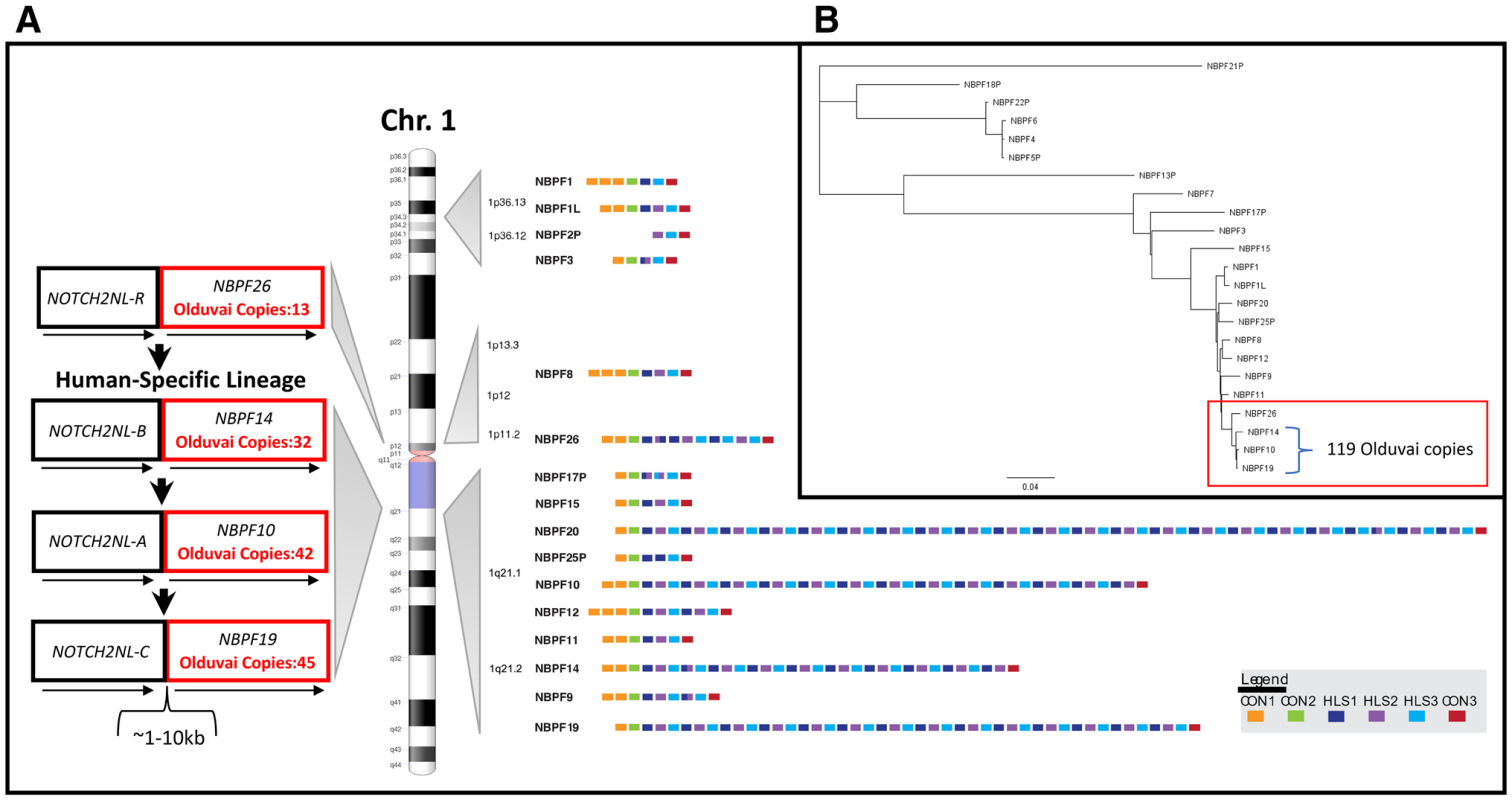
Genome organization, phylogeny, and recent evolution of human *NBPF* genes and Olduvai protein domains. **a** Shown to the right of chromosome 1 are the genome positions of *NBPF* genes and the number and arrangement of Olduvai domain subtypes based on the most recent human genome assembly (hg38) (Sikela and Quick 2018). Each block represents an Olduvai protein domain; each of the six primary Olduvai subtypes are depicted by a different color as denoted in the key. Shown to the left of chromosome 1 is the recent evolutionary history of *NOTCH2NL*/*NBPF* gene pairs and Olduvai protein domains. Model describes the order and timing of likely duplication events that produced multiple human-specific copies of *NOTCH2NL* and *NBPF* genes and human-specific hyper-amplification of Olduvai domains. Arrows below each gene indicate direction of orientation. **b** Phylogenetic profile of 23 human *NBPF* genes. Phylogenetic analysis was carried out on human *NBPF* genes. To avoid confusion resulting from the inclusion of highly similar Olduvai protein domains found in each *NBPF* gene, sequences were analyzed that only covered the start codon of each gene along with approximately 1 kb of flanking sequence. Phylogenetic profiling was performed using the Genious DNA analysis package and its multiple alignment program. Predicted number of Olduvai domains encoded by three human-specific *NBPF* genes (*NBPF10*, *NBPF14,* and *NBPF19*) is shown. The red box denotes the part of the *NBPF* phylogenetic profile which indicates that *NBPF26* is older than, and more evolutionarily distant from *NBPF10*, *NBPF14,* and *NBPF19*

The extreme human lineage-specific increase in Olduvai copy number has been almost entirely due to intragenic tandem expansions of a three-domain unit, termed the Olduvai triplet, that have occurred primarily in four human-specific *NBPF* genes (*NPBF10*, *NBPF14*, *NBPF19,* and *NBPF20*) that map to the 1q21.1-2 genomic region (O’Bleness et al. 2012a, b; O’Bleness et al. 2014) (Fig. 1a). Recently, three human-specific *NOTCH2NL* genes have been implicated in driving brain size in humans and also map to 1q21.1-2 (Fiddes et al. 2018; Suzuki et al. 2018; Florio et al. 2018). These studies provide evidence that the human-specific *NOTCH2NL* genes expand cortical progenitors and neuronal output and, thus, may have contributed to the expansion of the human cortex. More specifically, they propose that this occurs by increasing the level of self-renewal and/or expansion of radial glial cells, and could thereby facilitate an extended period of neurogenesis and the larger neuronal output that is characteristic of human corticogenesis. Here we provide genomic, phylogenetic, and transcriptional evidence that the new *NOTCH2NL* genes and adjacent Olduvai sequences may function in a coordinated manner to promote human brain expansion via a dosage-related mechanism.

## Methods

### Genomic and phylogenetic analysis

Genome coordinates for the locations of the *NBPF* and *NOTCH* genes were obtained from the hg38 human genome assembly at UCSC and also from several publications (O’Bleness et al. 2014; Sikela and Quick 2018; Fiddes et al. 2018; Suzuki et al. 2018). For phylogenetic analyses of *NBPF* genes, sequences were used that included the predicted start codon and 1 kb of sequence that flanked the start codon. This avoided complications that would arise from inclusion of the highly duplicated human Olduvai sequences, and allowed the analysis to focus specifically on the evolutionary relatedness of *NBPF* genes. Phylogenetic analysis of *NBPF* gene sequences was carried out with the Geneious Treebuilder program using the genetic distance model of Tamura-Nei (Tamura and Nei 1993) and the neighbor-joining tree build method (Geneious version 11.1 created by Biomatters. Available from https://www.geneious.com).

### Transcriptional analysis of correlations between *NBPF* and *NOTCH2NL* genes

Recently generated scRNA-seq data for human developing cortex were obtained from Nowakowski et al. (2017) available through the UCSC cell browser (https://cells.ucsc.edu/?ds=cortex-dev). Because reads mapping to *NOTCH2* and *NOTCHNL* transcripts were not well differentiated, we re-aligned reads to an aggregate *NOTCH2NL* gene model and to *NOTCH2* by mapping to the collapsed hg19 version of *NOTCH2NL*. We then performed a gene-by-gene Pearson correlation between *NOTCH2NL* expression and the profiles of all human genes detected across all cells (*n* = 22,988), and of all genes detected across radial glia only (*n* = 22,750).

For 3′ pileup studies, expression coverage tracks were obtained from the UCSC human cortex single-cell expression track hub for 572 radial glia cells. For each subtype of radial glia cells, the coverage track was normalized to sum to 1 and then multiplied by the number of cells in that subtype. The resulting cell and sequencing depth normalized coverage tracks were then summed to produce a final radial glial cell expression track.

## Results

### Genomic and phylogenetic evidence for paired evolution

Current human genomic data indicate that there are four human-specific *NOTCH2NL* genes: *NOTCH2NL*-*A*, *NOTCH2NL*-*B* and *NOTCH2NL*-*C*, located on 1q21.1, and *NOTCH2NL*-*R* located on 1p11.2 (Fiddes et al. 2018; Suzuki et al. 2018). While chimpanzee and gorilla have copies of *NOTCH2NL*, none are functional (Fiddes et al. 2018). Immediately adjacent to, and downstream of, each of these four *NOTCH* paralogs is an *NBPF* gene in the same orientation as its *NOTCH2NL* partner (O’Bleness et al. 2014; Fiddes et al. 2018; Suzuki et al. 2018) (Fig. 1a). This striking genomic arrangement suggests that each of the additional copies of *NOTCH2NL* that appeared in the human genome did not duplicate as a single gene, but rather did so as a two-gene module, composed of one *NOTCH2NL* gene and one *NBPF* gene.

While there remains some ambiguity about the precise steps by which the *NOTCH2NL* paralogs and adjacent *NBPF* genes appeared, several solutions have been proposed (Fig. 1a, Supplemental Fig. 1). Due to the rapid evolution of this region in both humans and other great apes, as well as the observed gene conversion between paralogs, it is difficult to conclusively determine which scenario happened. However, there are some lines of evidence in the human lineage that favor scenarios where *NOTCH2NL*-*A/*-*B/*-*C* derived from *NOTCH2L*-*R*. Because all *NOTCH2NL* genes in the human lineage are associated with *PDE4DIP*, and both chimpanzee and gorilla have one *PDE4DIP-*associated copy, the four human copies must derive from that copy. The previous analysis of the Simons Diversity Project (Fiddes et al. 2018) showed gene conversion between *NOTCH2NL*-*A* and *NOTCH2NL*-*B* is prevalent in the population, and gene conversion between *NOTCH2NL*-*C* and *NOTCH2NL*-*A/*-*B* happens infrequently. No gene conversion was observed between *NOTCH2* and *NOTCH2NL*-*R* and the three paralogs on 1q21.1. This is supported by the sequence identity observed between pairwise comparisons of *NOTCH2* and *NOTCH2NL* paralogs. In GRCh38, *NOTCH2* has 98.19% sequence similarity to *NOTCH2NL*-*R*, while *NOTCH2NL*-*A/*-*B/*-*C* have 98.89% to 98.91% identity to each other.

These lines of evidence suggest that a likely model for *NOTCH2NL* evolution in hominids involves first a gene conversion event between *NOTCH2NL*-*R* and *NOTCH2* restoring a functional 5′ end, and then, a subsequent duplication event sometime either shortly before or after the pericentric inversion of chromosome 1 (Yunis and Prakash 1982). Following this event, *NOTCH2NL*-*R* ceased gene converting with *NOTCH2,* while the new *NOTCH2NL* in 1q21.1 quickly gave rise to the other two paralogs. These three paralogs continue to have segregating gene conversion events happening to this day, which makes determining the exact order of their appearance difficult. Further supporting this model is that in the human assembly GRCh38 *NOTCH2NL*-*R* is 1.8% diverged from *NOTCH2*, while *NOTCH2NL*-*A/*-*B/*-*C* are 1.1% diverged, representing 61.1% fewer accumulated mutations, which is in line with expectation if the three loci are undergoing continual gene conversion and as a result evolving together.

If the four human-specific *NOTCH2NL* genes and four adjacent human-specific *NBPF* genes duplicated together as two-gene units, the phylogenetic history of these two sets of genes should be identical. To examine the relationships among the four *NBPF* genes that are paired with *NOTCH2NL* genes, we focused on sequence near the start codon that did not contain repeated Olduvai domains and was, therefore, less likely to be subject to gene conversion. These comparisons indicate that the three *NBPF* genes on 1q21.1-2 (*NBPF10*, *NBPF14*, and *NBPF19*) are more similar to one another than they are to *NBPF26* on 1p12 (Fig. 1b). In addition, *NBPF26* is the most related to ancestral *NBPF* genes, suggesting that the most plausible history involves *NOTCH2NL*-*R/NBPF26* being the original *PDE4DIP*-associated copy that survived from the most recent common ancestor with chimpanzee. Taken together, the data indicate that the *NOTCH2NL*-*R/NBPF26* gene pair on 1p12 duplicated as a unit, with the resulting new pair (*NOTCH2NL*-*B/NBPF14*) moving to the 1q21.1 region (Fig. 1a). Subsequently, *NOTCH2NL*-*B/NBPF14* duplicated two more times in the 1q21.1-2 region, generating *NOTCH2NL*-*A/NBPF10* and *NOTCH2NL*-*C/NBPF19*.

A striking difference becomes evident, however, when one compares the human-specific *NOTCH2NL*/*NBPF* gene increases with the Olduvai increases encoded by these *NBPF* genes. While the *NOTCH2NL* paralogs (and their *NBPF* partners) went from one gene to four in humans, Olduvai copies encoded by these *NBPF* genes underwent human-specific hyper-amplification, increasing from 13 copies (encoded by *NBPF26)* to 132 (i.e., adding 119 copies encoded by *NBPF10*, *NBPF14,* and *NBPF19*) (Fig. 1a, b).

The two most plausible scenarios for how these expansions occurred are the following: The long tandem Olduvai expansions found on *NBPF10*, *NBPF14,* and *NBPF19* either had to occur independently on each of the three new human-specific *NBPF* genes after the genes appeared in 1q21.1-2, or a duplicated copy of *NBPF26* appeared in 1q21.1-2 and, after adding many Olduvai copies (i.e., becoming *NBPF14*), duplicated twice more (i.e., producing *NBPF10* and *NBPF19*). The latter scenario is the most parsimonious and if, true, implies that each time one of these two new expanded duplicate genes appeared it would have instantaneously added large numbers of Olduvai copies to the human genome. In either case, the human-specific Olduvai hyper-amplification would have had to take place very recently (within the past 3 million years as has been proposed for the three *NOTCH2NL* genes on 1q21.1) and, thus, would correlate with the extreme enlargement of the human brain that occurred during this time (e.g., a threefold increase in size over the past 1.8 million years) (Florio et al. 2017).

### Transcriptional evidence for coordinated regulation

**Fig. 2:**
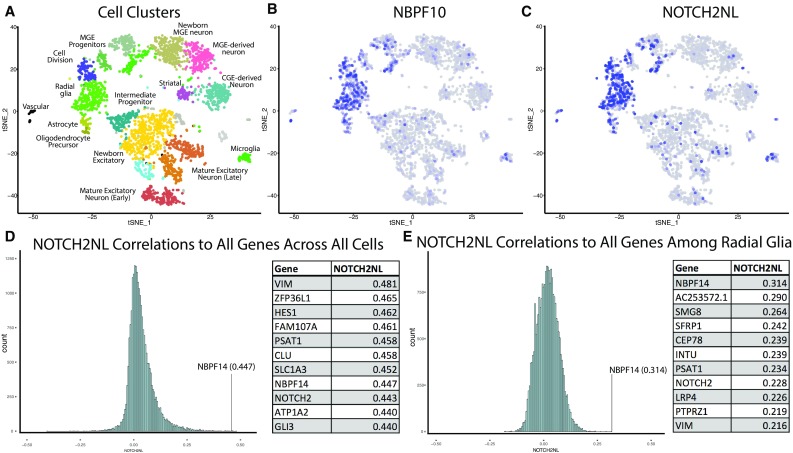
Radial glia-specific expression of *NBPF10* and *NOTCH2NL* in developing human telencephalon samples. **a**–**c** Scatterplot of 3412 cells from developing telencephalon after single-cell RNA-seq principal component analysis and t-stochastic neighbor embedding (tSNE) as described in Nowakowski et al. (2017). Cells are colored by annotated cell type clusters (**a**), *NBPF10* expression (**b**), and *NOTCH2NL* expression (**c**). Histograms of Pearson correlation coefficients are shown representing results of a comparison of scRNA-seq expression profiles of *NOTCH2NL* vs. all human genes from all cells (**d**) and from radial glia (**e**) with tables showing the top correlated genes

The fact that the three expanded human-specific *NBPF* genes are adjacent to (within 1–10 kb), and in the same orientation as, the three human-specific *NOTCH2* *NL* genes, raises the possibility that the expression of the *NBPF* genes and *NOTCH2NL* genes may be under shared transcriptional control. This possibility is further supported by transcriptional profiling using scRNA-seq (Pollen et al. 2014; Nowakowski et al. 2017), which indicates that *NBPF10* and *NOTCH2NL* show very similar expression patterns in developing human cortex, with especially high expression in radial glial cells (Fig. 2a–c). Radial glia are thought to be central contributors to neurogenesis and brain expansion (Hansen et al. 2010; Pollen et al. 2015). To more precisely examine whether *NOTCH2NL* and *NBPF* genes could be regulated by common mechanisms, we first quantified *NOTCH2NL* expression separately from *NOTCH2* by realigning single-cell reads to an aggregate gene model of *NOTCH2NL* paralogs (methods). We then examined the Pearson correlation of *NOTCH2NL* to all genes (*n* = 22,988) across diverse cortical cell types. The top correlates of *NOTCH2NL* included canonical radial glia markers such as *VIM*, *HES1,* and *SLC1A3* (Pollen et al. 2015), as well as *NBPF14, NOTCH2,* and *NBPF10* (Fig. 2d; Table S1). These strong correlations could reflect shared regulatory control, but they might simply reflect specificity for radial glia and the shared absence of expression in other cell populations. Therefore, we further examined the correlation of *NOTCH2NL* to all genes among radial glia cells only. Remarkably, the top gene correlated with *NOTCH2NL* out of all genes was *NBPF14* (Fig. 2e, Table S1). Therefore, *NOTCH2NL* and *NBPF* genes not only share radial glia specificity, but are also very tightly co-expressed at the level of single cells. This extremely strong correlation could be driven by a single fusion transcript recently observed with a *NOTCH2NL* paralog at the 5′ end and an *NBPF* paralog at the 3′ end (Dougherty et al. 2018). However, we see limited transcriptional evidence for this combined transcript as 3′ biased scRNA-seq reads are concentrated at the 3′ end of *NOTCH2NL*, suggesting that the majority of *NOTCH2NL* transcripts terminate as predicted (Supplementary Fig. 2A–C). Consistent with shared transcriptional regulation, the correlation between *NOTCH2NL* and *NBPF* genes is further supported across adult human tissues (Supplementary Fig. 2D). Notably, expression of these genes is relatively low in the adult brain compared with other tissue types and with radial glia in the developing cortex. These findings are consistent with other expression studies which show that *NBPF* (i.e., Olduvai) sequences are expressed at the right time and place to be important to cortical neurogenesis (Keeney et al. 2015), and are among a small set of human-specific genes that show high expression in neural progenitor cells (Suzuki et al. 2018; Florio et al. 2018).

## Discussion

The most probable model of how the four human-specific *NBPF/NOTCH2NL* gene pairs appeared in the human genome (Fig S1C) not only fits well with available phylogenetic data, but also may help explain the existence of two well-established cytogenetic landmarks associated with human chromosome 1: the human-specific pericentric inversion and the human-specific C-band at 1q12. In this model, the *NPBF26/NOTCHNL*-*R* gene pair appeared on 1p12 and then duplicated forming *NBPF14/NOTCH2NL*-*B*, which was then moved to the 1q21.1-2 region as a result of the human-specific pericentric inversion event. This placed *NBPF14/NOTCH2NL*-*B* next to the large human-specific band of constitutive heterochromatin (C-band) at 1q12. The C-band is rich with highly repeated sequences and a strong promoter of non-allelic homologous recombination (NAHR) events. Thus, proximity to the C-band could have driven both the production of the two additional human-specific 1q21.1 copies *(NBPF14/NOTCH2NL*-*A* and *NBPF19/NOTCH2NL*-*C)*, as well as the extreme expansions of the human-specific tandemly repeated copies of Olduvai that are found in *NBPF10*, *NBPF14,* and *NBPF19).*

In addition to these three expanded *NBPF* genes, there is a fourth gene in 1q21.1-2, *NBPF20*, that also contains long tandemly arranged human-specific copies of Olduvai. However, *NBPF20* does not have an adjacent *NOTCH2NL* gene and, while the other expanded *NBPF* genes (*NBPF10*, *NBPF14,* and *NBPF19*) show high sequence relatedness to one another, *NBPF20* is phylogenetically more distant (Fig. 1b). Given these findings, it is likely that the intragenic Olduvai expansions found on *NBPF20* arose independently, separate from the expansions found on *NBPF10*, *NBPF14,* and *NBPF19*. However, the fact that all four of the most highly expanded human-specific *NBPF* genes are found in the 1q21.1 region (and no highly expanded *NBPF* genes are found outside of this region) is also consistent with the possibility that proximity to the human-specific C-band at 1q12 may have promoted these extreme tandem expansions of Olduvai copy number in humans.

The tandemly expanded Olduvai sequences are found typically as Olduvai triplets in *NBPF10*, *NBPF14*, *NBPF19,* and *NBPF20* genes and, while triplet sequences are different between these genes, they are highly similar within each gene. This is likely the result of gene conversion and concerted evolution, which tends to homogenize tandemly repeated sequences within a gene (Ganley and Kobayashi 2007). In addition, the fact that, within an *NBPF* gene, the triplet sequences, as a unit, are more highly conserved than the individual domains supports the idea that homogenization is occurring at the level of the triplets within a gene.

While the 1q21 region is enriched for human-specific Olduvai sequences, it has also been linked with a number of disease-associated copy number variations. Among these, 1q21-associated duplications have been implicated in autism and macrocephaly, while reciprocal deletions have been linked with schizophrenia and microcephaly (Dumas et al. 2012; Brunetti-Pierri et al. 2008; Girirajan et al. 2013; Mefford et al. 2008; Stefansson et al. 2008). It has been suggested that the *NOTCH2NL*-*A* and *NOTCH2NL*-*B* genes provide the breakpoints for this syndrome, and, thus, have been instrumental in the recombination events that led to these gains/losses (Fiddes et al. 2018). However, because of the highly duplicated nature of the Olduvai sequences encoded by the adjacent *NBPF* genes (*NBPF10* and *NBPF14*), the involvement of Olduvai sequences in providing the breakpoints for this 1q21-associated duplication/deletion syndrome has not yet been directly examined. Given that highly duplicated, tandemly arranged sequences, such as the Olduvai repeats on *NBPF10* and *NBPF14*, are especially prone to recombination, it is likely that, in some cases, the breakpoints are within these *NBPF* genes. Higher resolution analyses may be able to more definitively address this issue in the future.

Because human-specific Olduvai sequences are found primarily as long tandemly arranged repeats, one might suspect that they serve only to promote recombination and, thus, are functionally unimportant. While such a recombinogenic architecture may have been instrumental in the generation of the newly duplicated *NOTCH2NL* paralogs and, as mentioned above, may be a significant contributor to the 1q21-duplication/deletion syndrome, there are several reasons why it is unlikely that Olduvai sequences are functionally silent partners of the *NOTCH2NL* genes. The Olduvai sequences found in the three *NBPF* genes adjacent to the *NOTCH2NL* genes encode long open-reading frames (O’Bleness et al. 2012a, b; O’Bleness et al. 2014) express a protein product that in brain is restricted to neurons (Popesco et al. 2006), and show strong signals of positive selection at the protein sequence level (Popesco et al. 2006). Functional data also support a more active role for Olduvai sequences in corticogenesis, as introduction of Olduvai sequences has been shown to promote proliferation of neural stem cells (Keeney et al. 2015). These observations, when combined with the findings reported here, suggest that human-specific Olduvai domains and adjacent *NOTCH2NL* genes may function in a coordinated, complementary fashion to promote neurogenesis and brain expansion in a dosage-related manner.

## Electronic supplementary material

Below is the link to the electronic supplementary material.

Supplementary Fig. S1. Possible *NBPF/NOTCH2NL* phylogenies. Three possible phylogenies are presented in increasing order of likelihood. A *NBPF*/*NOTCH2NL* pair present in 1p12 is moved to 1q21.1 via pericentric inversion. This copy is then duplicated back to 1p12 in addition to being duplicated further in 1q21.1. B *NBPF*/*NOTCH2NL* in 1p12 is duplicated to 1q21.1 prior to the pericentric inversion, then the inversion swaps the two copies. C *NBPF*/*NOTCH2NL* is duplicated a second time in 1p12 prior to the pericentric inversion, which moves only one of the two copies to 1q21.1 where it duplicates further. Scenario C is most likely because it involves a linear phylogeny, which is supported by the data (PDF 811 kb)

Supplementary Fig. S2.Normalized read coverage for *NOTCH2NL-A/-B/-C* and *NBPF10/14/19* was analyzed in 572 radial glia cells from a scRNA-seq method with a parital 3′ bias (A–C). A large spike in coverage is observed past the 3′ UTR of *NOTCH2NL* suggesting that expression of *NOTCH2NL* and *NBPF* mainly occurs through two separate transcripts in this cell type. D. Heatmap of gene expression values expressed as transcripts per million reads (TPM) across adult human tissue samples from the GTEx resource (https://gtexportal.org). Genes are clustered by expression across tissue types, and tissues are clustered based on expression of labeled genes. Because the dataset does not include *NBPF19* and *NBPF26*, only *NBPF10* and *NBPF14* are shown (PDF 456 kb)

Table data uploaded as Auxiliary Supplementary Materials. Table S1 contains Pearson correlation coefficients of the brain expression profiles of *NOTCH2* and *NOTCH2NL* vs. all genes (*n* = 22,988) among all cells (first two columns) and among radial glia only (third and fourth columns, *n* = 22,750 genes) (XLSX 1442 kb)
