# Supplementary figures and images for: Paired involvement of human-specific Olduvai domains and NOTCH2NL genes in human brain evolution
Source: Hum Genet. 2019 May 13;138(7):715–21. doi: 10.1007/s00439-019-02018-4 (PMC6611739; doi:10.1007/s00439-019-02018-4)

A)

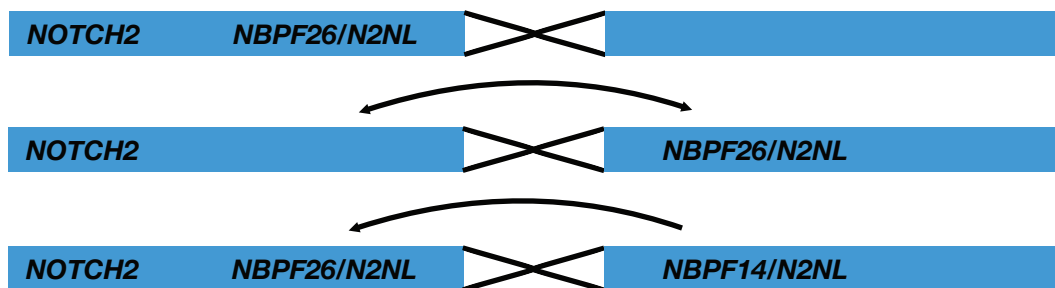

B)

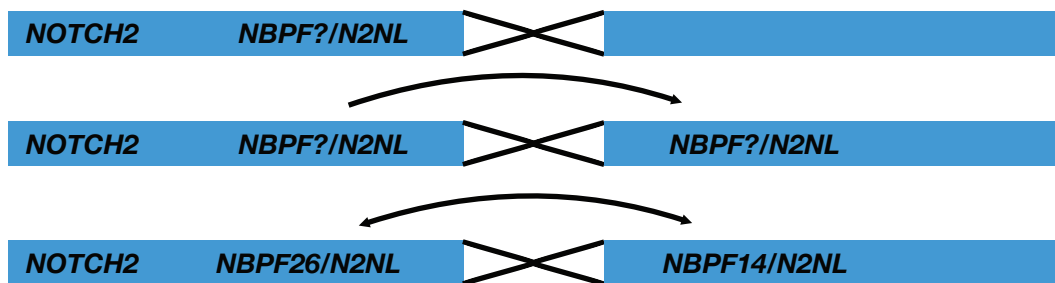

C)

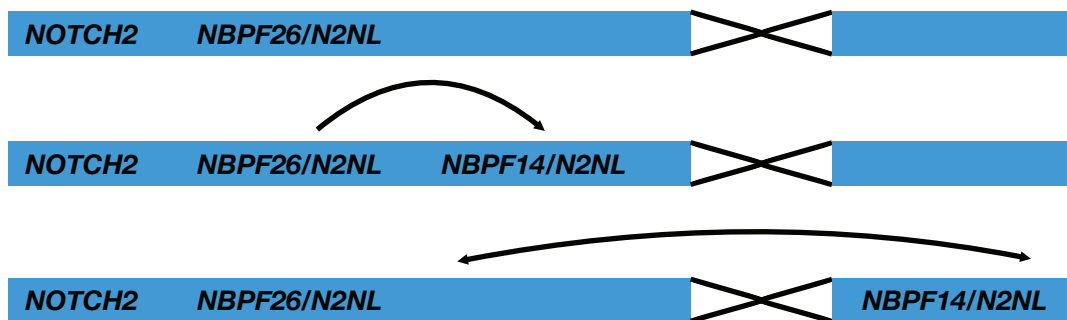

Supplement: Supplementary file 1 — Supplementary Fig. S1. Possible NBPF/NOTCH2NL phylogenies. Three possible phylogenies are presented in increasing order of likelihood. A NBPF/NOTCH2NL pair present in 1p12 is moved to 1q21.1 via pericentric inversion. This copy is then duplicated back to 1p12 in addition to being duplicated further in 1q21.1. B NBPF/NOTCH2NL in 1p12 is duplicated to 1q21.1 prior to the pericentric inversion, then the inversion swaps the two copies. C NBPF/NOTCH2NL is duplicated a second time in 1p12 prior to the pericentric inversion, which moves only one of the two copies to 1q21.1 where it duplicates further. Scenario C is most likely because it involves a linear phylogeny, which is supported by the data (PDF 811 kb) [file 439_2019_2018_MOESM1_ESM.pdf]

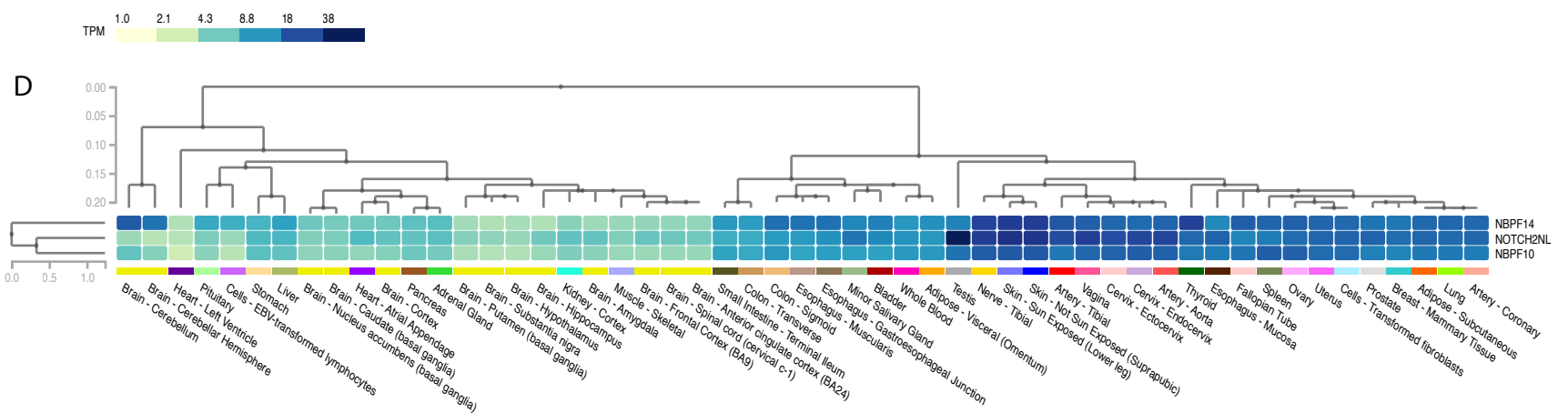

Supplement: Supplementary file 2 — Supplementary Fig. S2.Normalized read coverage for NOTCH2NL-A/-B/-C and NBPF10/14/19 was analyzed in 572 radial glia cells from a scRNA-seq method with a parital 3′ bias (A–C). A large spike in coverage is observed past the 3′ UTR of NOTCH2NL suggesting that expression of NOTCH2NL and NBPF mainly occurs through two separate transcripts in this cell type. D. Heatmap of gene expression values expressed as transcripts per million reads (TPM) across adult human tissue samples from the GTEx resource (https://gtexportal.org). Genes are clustered by expression across tissue types, and tissues are clustered based on expression of labeled genes. Because the dataset does not include NBPF19 and NBPF26, only NBPF10 and NBPF14 are shown (PDF 456 kb) [file 439_2019_2018_MOESM2_ESM.pdf]
